# “Weekend effect” on stroke mortality revisited: Application of a claims-based stroke severity index in a population-based cohort study

**DOI:** 10.1097/MD.0000000000004046

**Published:** 2016-06-24

**Authors:** Cheng-Yang Hsieh, Huey-Juan Lin, Chih-Hung Chen, Chung-Yi Li, Meng-Jun Chiu, Sheng-Feng Sung

**Affiliations:** a Department of Neurology, Tainan Sin Lau Hospital; b Department of Neurology, Chi Mei Medical Center; c Department of Neurology, National Cheng Kung University Hospital, College of Medicine, National Cheng Kung University; d Department of Public Health, College of Medicine, National Cheng Kung University, Tainan; e Department of Public Health, China Medical University, Taichung; f Department of Public Health, College of Medicine, Tainan; g Division of Neurology, Department of Internal Medicine, Ditmanson Medical Foundation Chiayi Christian Hospital, Chiayi City; h Department of Nursing, Min-Hwei Junior College of Health Care Management, Tainan, Taiwan.

**Keywords:** claims data, ischemic stroke, mortality, outcomes research, weekend

## Abstract

Previous studies have yielded inconsistent results on whether weekend admission is associated with increased mortality after stroke, partly because of differences in case mix. Claims-based studies generally lack sufficient information on disease severity and, thus, suffer from inadequate case-mix adjustment. In this study, we examined the effect of weekend admission on 30-day mortality in patients with ischemic stroke by using a claims-based stroke severity index.

This was an observational study using a representative sample of the National Health Insurance claims data linked to the National Death Registry. We identified patients hospitalized for ischemic stroke, and examined the effect of weekend admission on 30-day mortality with vs without adjustment for stroke severity by using multilevel logistic regression analysis adjusting for patient-, physician-, and hospital-related factors. We analyzed 46,007 ischemic stroke admissions, in which weekend admissions accounted for 23.0%. Patients admitted on weekends had significantly higher 30-day mortality (4.9% vs 4.0%, *P* < 0.001) and stroke severity index (7.8 vs 7.4, *P* < 0.001) than those admitted on weekdays. In multivariate analysis without adjustment for stroke severity, weekend admission was associated with increased 30-day mortality (odds ratio (OR), 1.20; 95% confidence interval [CI], 1.08–1.34). This association became null after adjustment for stroke severity (OR, 1.07; 95% CI, 0.95–1.20).

The “weekend effect” on stroke mortality might be attributed to higher stroke severity in weekend patients. While claims data are useful for examining stroke outcomes, adequate adjustment for stroke severity is warranted.

## 1 Introduction

It remains controversial whether patients with stroke admitted on weekends have a higher risk of mortality than those admitted on weekdays.^[1]^ Some previous studies have suggested that patients presenting with stroke on weekends have higher short-term mortality.^[2,3]^ Such “weekend effect” may be due to decreased staffing of healthcare workers and/or reduced access to emergency treatment for stroke on weekends. On the contrary, other studies have found that patients presenting with ischemic stroke on weekends have a higher chance of receiving intravenous thrombolytic therapy^[4–7]^—an emergency and standard treatment for acute ischemic stroke. Because some patients may have a higher stroke severity threshold for seeking health care on weekends than on weekdays,^[4,8]^ the weekend effect on stroke mortality may disappear after adjusting for stroke severity.^[9,10]^

Although administrative claims data typically lack detailed clinical information, they reflect routine clinical practice, and can be used as a set of proxies that indirectly represent the health status of patients.^[11]^ A previous study based on a national claims database in Taiwan indicated that weekend admission is associated with an approximately 20% higher adjusted odds of 30-day mortality in patients with ischemic stroke.^[12]^ However, the effect of weekend admission on mortality was not adjusted for stroke severity. Because of the heterogeneous size and location of vascular lesions, stroke severity varies greatly among stroke patients. Ideally, stroke severity should be evaluated with a clinical neurological scale, such as the National Institutes of Health Stroke Scale (NIHSS). Nevertheless, such clinical scales are generally unavailable in claims data. Therefore, lack of adjustment for stroke severity is commonly seen in claims-based stroke studies.^[13–15]^ This deficiency is particularly relevant for stroke outcomes research because stroke severity is a major determinant of stroke outcomes.^[16,17]^ To overcome this inherent shortcoming, we have developed a 7-item claims-based stroke severity index (SSI), which correlates well with initial stroke severity as assessed by using the NIHSS.^[18]^ This novel index has been satisfactorily applied to estimate stroke severity in patients hospitalized for ischemic stroke in our other claims-based study.^[19]^

The aim of the present study was to reexamine the effect of weekend admission on 30-day mortality in patients with ischemic stroke by using the SSI as a proxy for stroke severity. Our hypothesis was that the weekend effect on mortality may be attributable to stroke severity rather than other patient-, physician-, or hospital-related factors.

## 2 Methods

### 2.1 Data source

This was a retrospective cohort study conducted using Taiwan's National Health Insurance (NHI) claims data. The study protocol was approved by the Institutional Review Board of National Cheng Kung University Hospital (IRB No. B-EX-104-007). In brief, in Taiwan, a single-payer, mandatorily enrolled NHI program was launched in 1995 to provide universal coverage for inpatient care, outpatient care, dental care, and prescription medications. Large computerized datasets derived from this program are released for research purposes. In the present study, we used the Longitudinal Health Insurance Database (LHID) provided by the Health and Welfare Statistics Application Center of the Ministry of Health and Welfare. The LHID contains all the registration and claims data from 2000 to 2013 of 2 million individuals randomly sampled from the 23.8 million NHI enrollees in 2000. Up to 5 International Classification of Diseases, Ninth Revision, Clinical Modification (ICD-9-CM) diagnosis codes are listed on inpatient claims, and up to 3 ICD-9-CM diagnosis codes are listed on outpatient claims. There were no significant differences in the distribution of age, sex, or cause of death between subjects in the LHID and those in the original claims database for all enrollees.^[20]^

### 2.2 Study cohort

We identified consecutive patients who were hospitalized to acute care hospitals between January 1, 2001 and December 1, 2013, with a principal discharge diagnosis of ischemic stroke, defined as an ICD-9-CM code of 433.xx or 434.xx. The accuracy of ICD-9-CM coding for ischemic stroke in the NHI claims data has been validated with a sensitivity of 94.5% to 97.3% and a positive predictive value of 88.4% to 97.8%.^[21,22]^ Two or more admissions within 30 days in a single patient were considered to be the same stroke episode, and only the first admission was used as the index admission for analysis. The date of the index admission was designated as the index date.

### 2.3 Study variables

The outcome of interest was all-cause mortality within 30 days after the index date. We linked patient files in the LHID to the government National Death Registry by using a unique patient identifier to determine patient vital status after stroke. All patients were followed up to their death or to the 30-day endpoint, whichever came first.

The primary independent variable was admission on weekends (Saturday and Sunday) vs weekdays (Monday through Friday). Patient-related covariates included year of the index date, age, sex, brain surgery, intravenous thrombolysis, a modified version of the Charlson comorbidity index (CCI),^[23]^ and the SSI. We retrieved all diagnosis codes from the inpatient and outpatient claims during a 12-month baseline period before the index date to calculate the CCI. The SSI comprises 7 claims items, including airway suctioning, bacterial sensitivity test, general ward stay, intensive care unit stay, nasogastric intubation, osmotherapy, and urinary catheterization.^[18]^ We determined presence of these items from the inpatient claims data of the index admission, and entered the results into a multiple linear regression equation (Table 1) to obtain the SSI.^[18]^

**Table 1:**
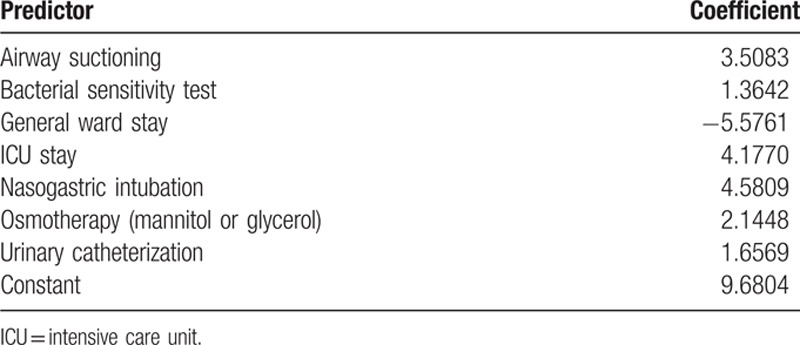
Multiple linear regression model for the stroke severity index.^[18]^

Based on a prior study,^[12]^ physician-related factors included physician specialty (neurology or other) and physician volume (number of stroke patient services per year). Hospital-related factors included hospital ownership (for profit, public, or not for profit), accreditation level (academic medical center, regional, or district), hospital volume (number of stroke patient admissions per year), location (Taipei, northern, central, southern, Kao-Ping, or eastern), and in-area bed supply (number of beds per 10,000 enrollees).

### 2.4 Statistical analysis

Continuous variables were summarized as means and standard deviations, while categorical variables were summarized as counts and percentages. Variables were compared between groups by using *t* tests for continuous variables and Chi-square tests for categorical variables. Pearson correlations were used to examine any relationship that may exist between stroke severity as assessed by using the SSI, number of admissions, as well as mortality by day of the week.

Because data on health care of patients and outcomes have a multilevel structure, assessment of health outcomes should account for the clustering effect of patients by physicians and hospitals.^[24]^ For example, patients treated by a physician may share specific characteristics. Similarly, patients admitted to a hospital may resemble each other but differ from those admitted to another hospital. Previous studies suggested that hierarchical generalized linear models are suitable for such multilevel structured data and have the potential of avoiding false inferences.^[25]^ Therefore, we performed multilevel logistic regression analysis (a hierarchical generalized linear model) to explore the association of weekend admission and 30-day mortality with or without including SSI as a covariate, and with adjustment for other patient-, physician-, and hospital-related factors. The data were structured as patient admissions (level 1) nested within physicians (level 2), who were in turn nested within hospitals (level 3).

All statistical analyses were performed using SAS version 9.3 software (SAS Institute, Cary, NC). A 2-tailed *P*-value of <0.05 was considered statistically significant.

## 3 Results

During the study period, a total of 46,007 ischemic stroke admissions for 37,679 patients, who were cared for by 4282 physicians in 413 hospitals, were identified. The length of stay was a median of 9 days (interquartile range 7–16 days). Table 2 lists the characteristics of the study cohort. In brief, 23.0% of stroke admissions occurred on weekends, and overall 30-day all-cause mortality was 4.2%. Patients admitted on weekends had significantly higher mortality than those admitted on weekdays (4.9% vs 4.0%, *P* < 0.001). In addition, patients admitted on weekends had a significantly higher SSI than those admitted on weekdays (7.8 vs 7.4, *P* < 0.001). The figure shows the distribution of stroke admissions, SSI, and 30-day mortality according to day of the week. SSI positively correlated with 30-day mortality (Pearson correlation coefficient, 0.846; *P* = 0.016), and negatively correlated with the number of admissions (Pearson correlation coefficient, −0.981; *P* < 0.001). Patients admitted on Sunday had the highest SSI, followed by those admitted on Saturday, whereas patients admitted on Monday had the lowest SSI. In contrast, the number of admissions was lowest on Sunday, second lowest on Saturday, and peaked on Monday.

**Table 2:**
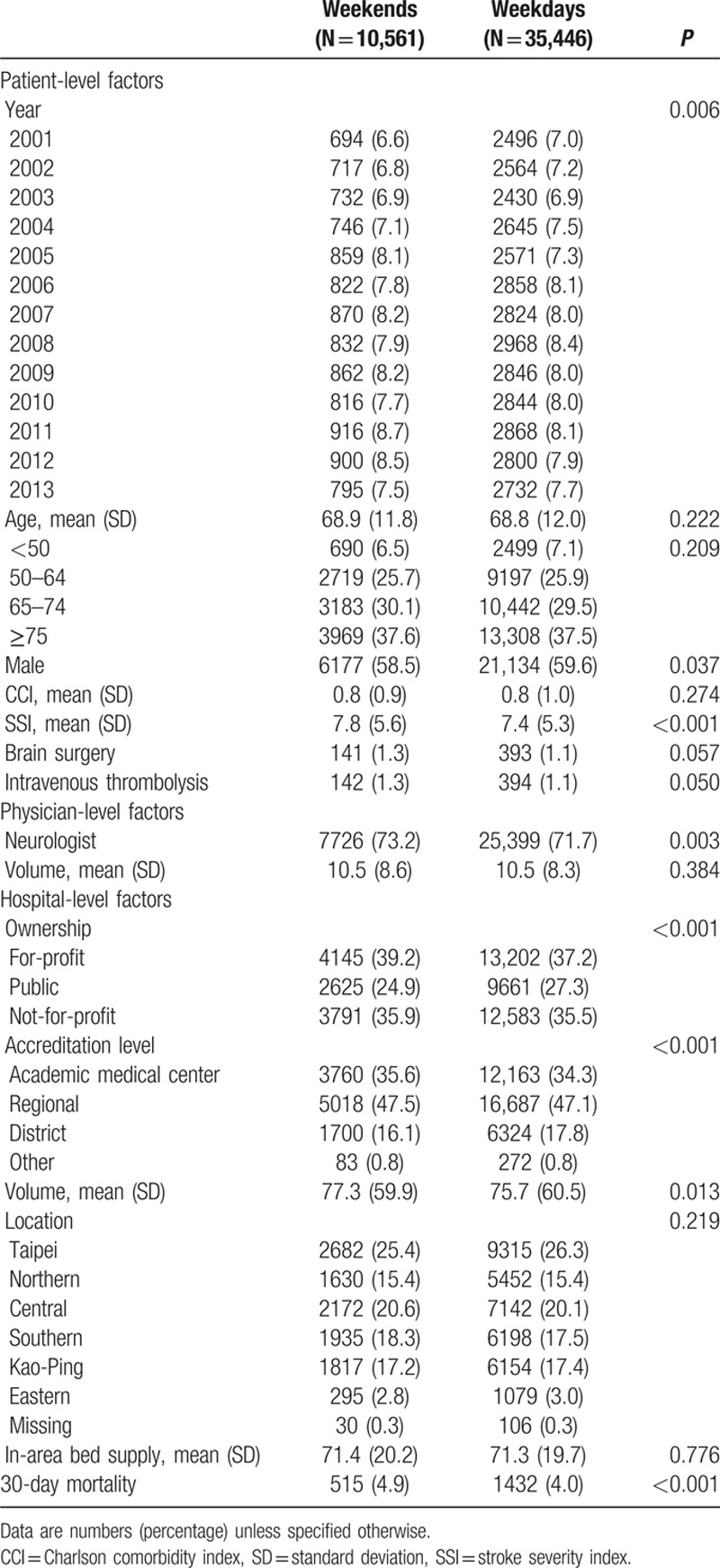
Characteristics of the study cohort (N = 46,007).

The unadjusted odds ratio (OR) of weekend admission on 30-day mortality was 1.19 (95% confidence interval [CI], 1.09–1.31). In multivariate analysis, the effect of weekend admission on mortality did not change materially in the model without adjustment for SSI (OR, 1.20; 95% CI, 1.08–1.34), but decreased and became statistically nonsignificant in the model with adjustment for SSI (OR, 1.07; 95% CI, 0.95–1.20) (Table 3). Older age and higher CCI carried a higher mortality risk regardless of adjustment for SSI. Notably, the harmful effect of brain surgery (OR, 3.46; 95% CI, 2.59–4.62) and intravenous thrombolysis (OR, 1.64; 95% CI, 1.12–2.41) became null (OR, 0.76; 95% CI, 0.57–1.01 for brain surgery) or protective (OR, 0.64; 95% CI, 0.43–0.95 for intravenous thrombolysis) after SSI was included in the model.

**Table 3:**
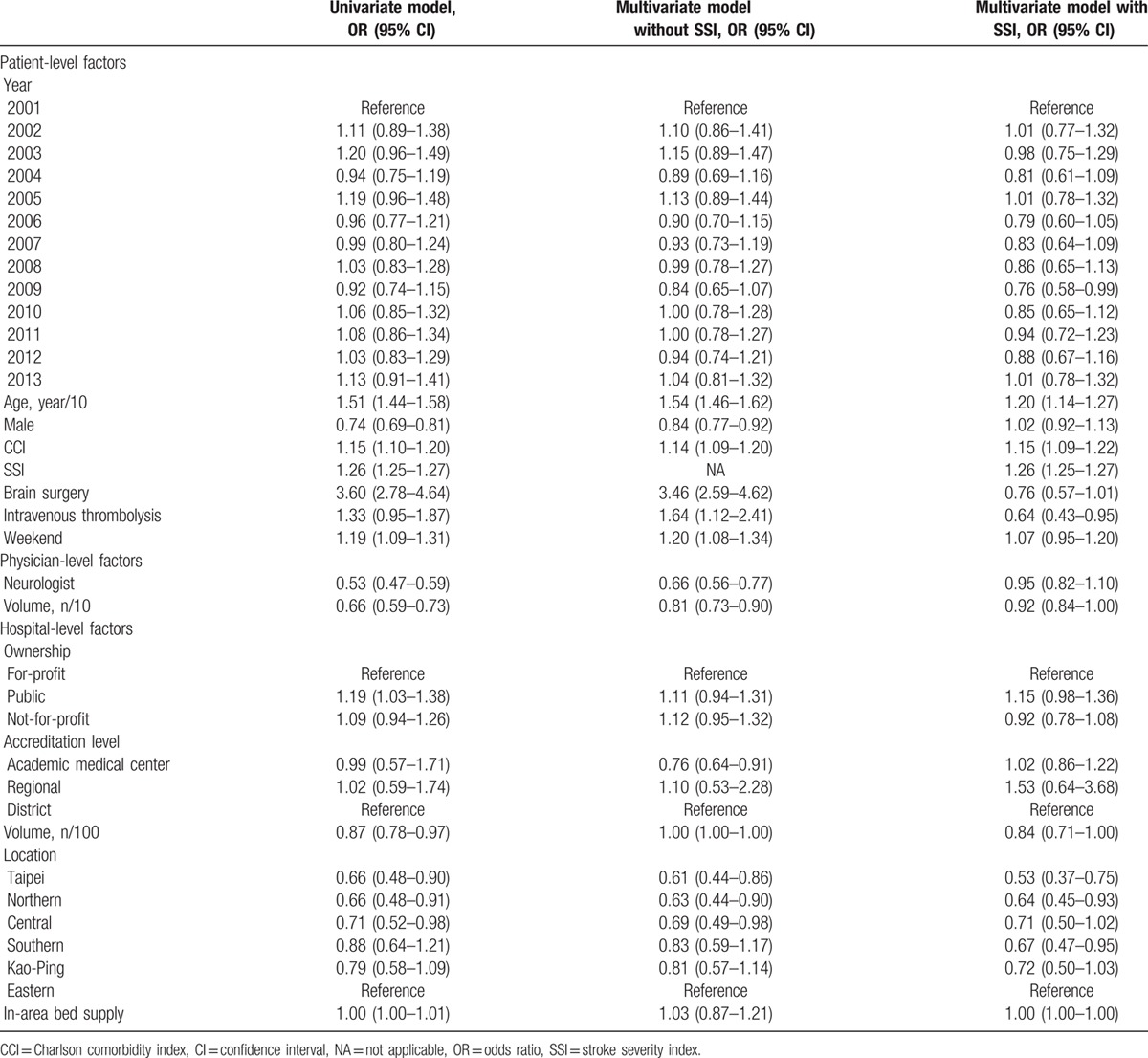
Multilevel logistic regression analysis of 30-day all cause mortality.

## 4 Discussion

We found that weekend admission for stroke was associated with higher 30-day all-cause mortality, but the association disappeared after adjustment for SSI, a claims-based proxy for stroke severity. The higher mortality for weekend admission was mainly due to patients admitted on weekends having higher stroke severity than those admitted on weekdays.

In addition to the weekend effect, we also noted a “Monday effect”; that is, the highest number of admissions and the lowest SSI were seen on Monday. Moreover, a strong negative correlation was found between SSI and the number of admissions by day of the week. This effect is probably because patients who experienced mild stroke on weekends tended to delay their admission until weekdays. In other words, the variations in the number of admissions across day of the week is mainly due to the shift of patients with lower stroke severity from weekends to weekdays.^[3]^ These observations are consistent with our clinical experience as well as prior studies, in which patients with mild stroke were more likely to delay their hospital arrival.^[26,27]^ The intentional delay in seeking health care by patients with stroke probably reflects their fear of lower quality of clinical service during weekends. Such different severity threshold for seeking care between weekends and weekdays also has been shown in other studies.^[3,4,8,10,28]^ The higher stroke severity in weekend patients and their shorter onset to arrival time might explain the increased use of intravenous thrombolytic therapy during weekends.^[3–7]^

**Figure 1:**
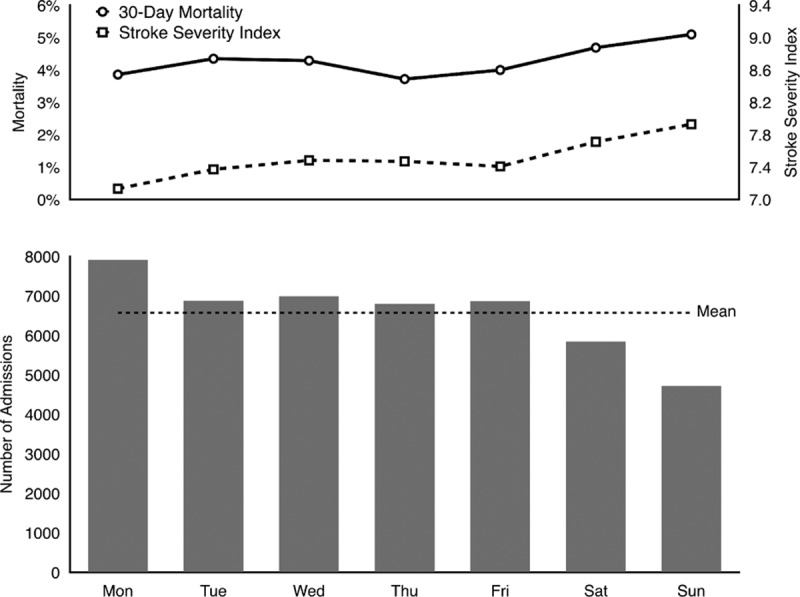
Number of admissions, mean stroke severity index, and 30-day mortality according to day of the week. The dashed line in the lower panel indicates the mean of admissions per day.

Even though the SSI correlates well with stroke severity,^[18]^ some concerns are raised because the SSI is not specific for evaluation of stroke severity and could be affected by complications of stroke. Actually, the rationale behind the development of the SSI lies in the observation that a higher stroke severity leads to more complications.^[18]^ The component items of the SSI reflect how these complications are treated and, consequently, a higher SSI value stands for a more severe stroke. It may be argued that poor care quality also results in complications, and thus increases the value of SSI. The high SSI on weekends might be due to low care quality during this period. However, if this is the case, then it is hard to explain the result of the lowest SSI on Monday (Fig. 1) because it is unlikely that patients admitted on Monday received a better quality of care than those admitted from Tuesday through Friday. Therefore, we believe that the high SSI on weekends truly represents high stroke severity although we are unable to rule out the possibility that care quality might play a minor role.

Increased mortality for weekend admission, or, more precisely, off-hour admission, in patients with stroke has been observed in studies that did not consider stroke severity.^[2,12,29–34]^ However, the effect of weekend or off-hour admission on mortality has been negated after adjustment for stroke severity as assessed by using a stroke scale or a similar proxy,^[4,9,10,28,35,36]^ as seen in the present study. The present analysis also found that the effect of brain surgery on mortality turned from harmful to null after adjustment for stroke severity. Such result is not surprising considering that surgical treatment (e.g., hemicraniectomy) may improve survival in patients with severe ischemic stroke.^[37]^ Similarly, the harmful effect of intravenous thrombolysis on mortality became protective when stroke severity was included in the model because intravenous thrombolysis was indicated for patients with more severe stroke (NIHSS ≥ 6) during the study period according to Taiwan's NHI coverage. The above-mentioned findings highlight the importance of adequate case-mix adjustment for stroke severity in stroke outcomes research, especially in studies based on claims data. Although information on disease severity is generally unavailable in claims data, the claims-based SSI may serve as a feasible proxy for stroke severity for investigators who wish to use Taiwan's NHI claims data in their stroke research.

Interestingly, in some studies, mortality after stroke was still higher with weekend or off-hour presentation even after adjustment for case mix and stroke severity.^[3,8]^ These results implicate that other factors in addition to stroke severity might still underlie the weekend effect. One previous study found unequal provision of stroke care between regular working hours and off-hours,^[8]^ whereas another study showed no significant differences in stroke care on weekends vs weekdays.^[3]^ Even though the higher mortality for weekend admission is largely explained by underlying stroke severity, efforts to improve quality of care should be continued and encouraged. Reorganization of stroke care to provide 24/7 access to stroke specialists, adequate staffing of nurses with stroke experience on weekends, and an organized system for delivering care may alleviate the weekend effect and save lives.^[9,32,38]^

This study has several limitations. First, we only focused on weekend days rather than all off-hours, when hospitals are likely to be understaffed. This was because the claims database we used in this study does not contain information on time of day. However, because patients admitted at off-hours, whether on weekends or during nighttime on weekdays, are more likely to have a severe stroke,^[30]^ we believe that the main finding of this study would not have changed if we had used a more extended definition of off-hours. Second, we did not consider the time from stroke onset to hospital arrival because data on this item was unavailable in the claims database. Although mortality might not be significantly affected by early or late presentation in unselected patients with acute stroke,^[39]^ functional outcomes and in-hospital mortality are time-dependent for those receiving intravenous thrombolysis.^[40,41]^ Third, important variables that might affect mortality, such as lifestyle and socioeconomic status, were also unavailable in the claims database. In addition, we did not have information on quality of stroke care. Fourth, because the SSI is based on the management and treatment provided for stroke patients during hospitalization, the differences in practice patterns across physicians and hospitals may influence the performance of the SSI. Nevertheless, the SSI has been shown to highly correlate with the admission NIHSS scores across cohorts from 4 hospitals of different sizes and types.^[18]^ Furthermore, we used multilevel modeling to account for the clustering effect among patients treated by different physicians in different hospitals, and thus could have minimized the effect of variation in practice patterns on the performance of the SSI. Fifth, the claims-based SSI has only been validated using Taiwan's NHI claims data. Further investigations are required to determine the applicability of the SSI as a proxy for stroke severity in claims databases from other healthcare systems. Nonetheless, a strength of this study is the population-based approach in identifying all patients seen at different types of hospitals. In addition, the vital status of patients was obtained from linkage with the National Death Registry, which minimized outcome measurement bias.

In conclusion, in Taiwan, patients with ischemic stroke admitted on weekends had higher 30-day mortality and SSI than those admitted on weekdays. The weekend effect on stroke mortality might be attributed to higher stroke severity in weekend patients. While claims data are useful for examining stroke outcomes, adequate adjustment for stroke severity is warranted.

## Acknowledgments

We would like to thank Editage (www.editage.com) and Ms. Li-Ying Sung for English language editing.
